# Supplementary material for: Caenorhabditis elegans methionine/S-adenosylmethionine cycle activity is sensed and adjusted by a nuclear hormone receptor
Source: eLife. 2020 Oct 5;9:e60259. doi: 10.7554/eLife.60259 (PMC7561351; doi:10.7554/eLife.60259)
Supplement: Transparent reporting form [file elife-60259-transrepform.pdf]

eLIFE
1st Floor
24 Hills Road
Cambridge CB2 1JP, UK
P 01223 855340
W elifesciences.org
T @elife

eLife's transparent reporting form

We encourage authors to provide detailed information *within their submission* to facilitate the interpretation and replication of experiments. Authors can upload supporting documentation to indicate the use of appropriate reporting guidelines for health-related research (see EQUATOR Network), life science research (see the BioSharing Information Resource), or the ARRIVE guidelines for reporting work involving animal research. Where applicable, authors should refer to any relevant reporting standards documents in this form.

If you have any questions, please consult our Journal Policies and/or contact us: editorial@elifesciences.org.

Sample-size estimation

- You should state whether an appropriate sample size was computed when the study was being designed
- You should state the statistical method of sample size computation and any required assumptions
- If no explicit power analysis was used, you should describe how you decided what sample (replicate) size (number) to use

Please outline where this information can be found within the submission (e.g., sections or figure legends), or explain why this information doesn't apply to your submission:

The RNA-seq dataset in Figure 1A, Figure 3C-D and Figure 6 is described in Bulcha *et al.*, 2019. Briefly, 3000 animals each were used for two biological replicates. Differential gene expression between samples was analyzed using the standard output of BGI bioinformatics pipeline using EBSeq (Leng *et al.*, 2013. EBSeq: an empirical Bayes hierarchical model for inference in RNA-seq experiments. *Bioinformatics* 29, 1035–43).

RNA-seq datasets Figure 3A-B, Figure 4D-E, Figure 5F-H and Figure 6 had a minimum of 400 animals per sample and were used for two biological replicates. For experiments in Figure 3A-B and Figure 4D-E, raw reads were processed on the DolphinNext RSEM v1.2.28 pipeline revision 7 (Yukselen *et al.*, 2020. DolphinNext: a distributed data processing platform for high throughput genomics. *BMC Genomics* 21, 310). For experiments in Figure 5F-H and Figure 6, read counts for each gene were used in differential expression analysis by DEseq2 package in R 3.6.3 (Love *et al.*, 2014. Moderated estimation of fold change and dispersion for RNA-seq data with DESeq2. *Genome Biol* 15, 550).

For the EMS screen, at least 8,000 haploid genomes were screened.

Primary RNAi screens were performed two independent times with about 15-20 animals in each trial, and hits were further tested an additional three times with about 200 animals in each trial unless otherwise stated.

Body size measurements in Figure 4C and Figure S1C include approximately 100 animals per sample.

GC-MS data in Figure 5A and 5E had roughly 3000 animals per sample.

Replicates

eLife Sciences Publications, Ltd is a limited liability non-profit non-stock corporation incorporated in the State of Delaware, USA, with company number 5030732, and is registered in the UK with company number FC030576 and branch number BR015634 at the address 1st Floor, 24 Hills Road, Cambridge CB2 1JP | August 2014

- You should report how often each experiment was performed
- You should include a definition of biological versus technical replication
- The data obtained should be provided and sufficient information should be provided to indicate the number of independent biological and/or technical replicates
- If you encountered any outliers, you should describe how these were handled
- Criteria for exclusion/inclusion of data should be clearly stated
- High-throughput sequence data should be uploaded before submission, with a private link for reviewers provided (these are available from both GEO and ArrayExpress)

Please outline where this information can be found within the submission (e.g., sections or figure legends), or explain why this information doesn't apply to your submission:

The RNA-seq dataset in Figure 1A, Figure 3C-D and Figure 6 is described in Bulcha *et al.*, 2019. Briefly, 3000 animals each were used for two biological replicates. The RNA-seq datasets in Figure 3A-B, Figure 4D-E, Figure 5F-H and Figure 6 had two biological replicates and a minimum of 400 animals per sample. Biological replicates are defined as independently growing, counting and collecting animals and obtaining the RNA from these animals all on different days.

The RNA-sequencing data files for Bulcha *at el.*, 2019, was deposited in the NCBI Gene Expression Omnibus (GEO) under accession number GSE123507, and for this study under GSE151848.

Bulcha: https://www.ncbi.nlm.nih.gov/geo/query/acc.cgi?acc=GSE123507
Geise: https://www.ncbi.nlm.nih.gov/geo/query/acc.cgi?acc=GSE151848

Primary RNAi screens were performed two biological times with about 15-20 animals in each trial, and hits were further tested an additional three biological times with about 200 animals in each trial unless otherwise stated. Biological replicates are defined as independently growing animals, harvesting them, and visually scoring them all on different days.

Body size measurements in Figure 4C and Figure S1C contain two biological replicates, with both replicates shown in the figures. Biological replicates are defined as independently growing animals, harvesting and imaging them, and computationally scoring the animal's body size all on different days.

GC-MS data as seen in Figure 5A and 5E have a minimum of four biological replicates. Biological replicates are defined as independently growing animals, harvesting them, extracting, derivatizing and performing the GC-MS all on different days.

eLife Sciences Publications, Ltd is a limited liability non-profit non-stock corporation incorporated in the State of Delaware, USA, with company number 5030732, and is registered in the UK with company number FC030576 and branch number BR015634 at the address 1st Floor, 24 Hills Road, Cambridge CB2 1JP | August 2014

Statistical reporting

- Statistical analysis methods should be described and justified
- Raw data should be presented in figures whenever informative to do so (typically when N per group is less than 10)
- For each experiment, you should identify the statistical tests used, exact values of N, definitions of center, methods of multiple test correction, and dispersion and precision measures (e.g., mean, median, SD, SEM, confidence intervals; and, for the major substantive results, a measure of effect size (e.g., Pearson's r, Cohen's d)
- Report exact p-values wherever possible alongside the summary statistics and 95% confidence intervals. These should be reported for all key questions and not only when the p-value is less than 0.05.

Please outline where this information can be found within the submission (e.g., sections or figure legends), or explain why this information doesn't apply to your submission:

Statistical analysis for RNA-seq can be found in the Materials and Methods. Raw data is available in GEO as mentioned above. In the figures RNA-seq data is represented as TPM (Transcripts per million reads) or log2 Fold Change. Figures 3C-D, Figure 4D, Figure 5F and 5H, and Figure 6A-D bar graphs represent the mean.

Body size measurement as seen in Figure 4C and Figure S1C shows individual data points for each animal and the bar represents the mean with standard deviation shown in brackets. Statistical significance was determined by the Kruskal-Wallis test with post hoc comparison using Dunn's multiple comparison test.

GC-MS data as seen in Figure 5A and 5E is plotted as standard boxplots showing the median and interquartile range. Statistical significance determined by two-tailed t-test for 5A and one-way ANOVA with post-hoc Dunnett's T3 test for 5E.

(For large datasets, or papers with a very large number of statistical tests, you may upload a single table file with tests, Ns, etc., with reference to sections in the manuscript.)

Group allocation

- Indicate how samples were allocated into experimental groups (in the case of clinical studies, please specify allocation to treatment method); if randomization was used, please also state if restricted randomization was applied
- Indicate if masking was used during group allocation, data collection and/or data analysis

Please outline where this information can be found within the submission (e.g., sections or figure legends), or explain why this information doesn't apply to your submission:

There was no group allocation in this project.

Additional data files ("source data")

- We encourage you to upload relevant additional data files, such as numerical data that are represented as a graph in a figure, or as a summary table
- Where provided, these should be in the most useful format, and they can be uploaded as "Source data" files linked to a main figure or table

- Include model definition files including the full list of parameters used
- Include code used for data analysis (e.g., R, MatLab)
- Avoid stating that data files are "available upon request"

Please indicate the figures or tables for which source data files have been provided:

For body size analysis as seen in Figure 4C and Figure S1C a MATLAB script named wormFinder.m was used and the source code is provided here
https://github.com/shiaway/wormFinder/blob/master/wormFinder.m
